# *RDH12* retinopathy: novel mutations and phenotypic description

**Published:** 2011-10-19

**Authors:** Donna S. Mackay, Arundhati Dev Borman, Phillip Moradi, Robert H. Henderson, Zheng Li, Genevieve A. Wright, Naushin Waseem, Mamatha Gandra, Dorothy A. Thompson, Shomi S. Bhattacharya, Graham E. Holder, Andrew R. Webster, Anthony T. Moore

**Affiliations:** 1 Department of Genetics, Institute of Ophthalmology, London, UK; 2 Moorfields Eye Hospital, London, UK; 3 SNONGC Department of Genetics & Molecular Biology, Vision Research Foundation, Sankara Nethralaya, Chennai, India; 4 Clinical and Academic Department of Ophthalmology, Great Ormond Street Hospital for Children, London, UK; 5 Department of Ophthalmology, Tongji Hospital and Medical College, Huazhong University of Science and Technology, Wuhan, China

## Abstract

**Purpose:**

To identify patients with autosomal recessive retinal dystrophy caused by mutations in the gene, retinal dehydrogenase 12 (*RDH12*), and to report the associated phenotype.

**Methods:**

After giving informed consent, all patients underwent full clinical evaluation. Patients were selected for mutation analysis based upon positive results from the Asper Ophthalmics Leber congenital amaurosis arrayed primer extansion (APEX) microarray screening, linkage analysis, or their clinical phenotype. All coding exons of *RDH12* were screened by direct Sanger sequencing. Potential variants were checked for segregation in the respective families and screened in controls, and their pathogenicity analyzed using in silico prediction programs.

**Results:**

Screening of 389 probands by the APEX microarray and/or direct sequencing identified bi-allelic mutations in 29 families. Seventeen novel mutations were identified. The phenotype in these patients presented with a severe early-onset rod-cone dystrophy. Funduscopy showed severe generalized retinal pigment epithelial and retinal atrophy, which progressed to dense, widespread intraretinal pigment migration by adulthood. The macula showed severe atrophy, with pigmentation and yellowing, and corresponding loss of fundus autofluorescence. Optical coherence tomography revealed marked retinal thinning and excavation at the macula.

**Conclusions:**

*RDH12* mutations account for approximately 7% of disease in our cohort of patients diagnosed with Leber congenital amaurosis and early-onset retinal dystrophy. The clinical features of this disorder are highly characteristic and facilitate candidate gene screening. The term *RDH12* retinopathy is proposed as a more accurate description.

## Introduction

Leber congenital amaurosis (LCA), first described by Theodor Leber in 1869 [1], is a heterogeneous autosomal recessive, generalized retinal dystrophy that presents at birth or soon after. The disorder is now recognized as the most severe form of a spectrum of early-onset retinal dystrophies (EORD), accounting for 3%–5% of childhood blindness in the developed world, with an estimated incidence of 2–3 per 100,000 live births [2]. Presentation is usually with reduced vision and nystagmus in early infancy. Undetectable or severely reduced rod and cone electroretinograms confirm the diagnosis [3,4]. The retinal appearance may initially be normal or show a variety of abnormalities, including white dots at the level of the retinal pigment epithelium (RPE), retinal pigment migration, retinal vascular attenuation, and macular atrophy.

To date, 14 causative genes, guanylate cyclase 2D (*GUCY2D*) [5], aryl hydrocarbon receptor interacting protein-like 1 (*AIPL1*) [6], retinal pigment epithelium-specific protein 65 (*RPE65*) [7], retinitis pigmentosa GTPase regulator interacting protein 1 (R*PGRIP1*) [8], cone-rod homeobox-containing gene (*CRX*) [9], tubby like protein 1 (*TULP1*) [10], crumbs homolog-1 (*CRB1*) [11], retinol dehydrogenase 12 (*RDH12*) [12], centrosomal protein 290 kDa (*CEP290*) [13], lebercilin (*LCA5*) [14], spermatogenesis-assoicated protein 7 (*SPATA7*) [15], lecithin retinol acyltransferase (*LRAT*) [16], c-mer proto-oncogene tyrosine kinase (*MERTK*) [17], and IQ motif-containing protein 1 (*IQCB1*) [18], and one more locus, *LCA9* [19]) have been identified. The *RDH12* gene, consisting of seven coding exons was identified due to sequence homology to *RD11* (originally named *PSDR1*) [20], and mapped to chromosome 14q23.3 [20,21]. *RDH12* mapped to the same region of chromosome 14 as two loci for LCA known as LCA3/LCA13 [22]. In 2004, the first mutations in families mapped to LCA13, were identified [12].

RDH12 expression is highest in the retina, where it localizes to the inner segments of rod and cone photoreceptors [21,23]. The protein sequence places it in the short chain dehydrogenase/reductase family. It was thought to be responsible for the conversion of vitamin A (all-trans retinal) to 11-cis retinal during the regeneration of cone visual pigments. But in the murine model, disruption of *RDH12* neither causes a retinal dystrophy nor affects the levels of all-trans and 11-cis retinoids [23]. It has been proposed that RDH12 functions to protect the retina from excessive all-trans retinal accumulation in continuous illumination [24,25]. There is some evidence, at least in the mouse retina, that RDH12 may be involved in detoxifying 4-hydroxynonenal in photoreceptor cells [26].

*RDH12* mutations have been associated with LCA [27,28], EORD [12], and with one family of autosomal-dominant retinitis pigmentosa [29]. Published phenotypic data suggests that visual symptoms first develop in early childhood. There is subsequent disease progression with extensive photoreceptor cell loss by adulthood [12,30-32]. Fundus examination at that stage shows a severe pigmentary retinopathy, with macular atrophy and vascular attenuation [12,30-33]. Electroretinographic findings reveal severe generalized loss of rod and cone photoreceptor function.

Here, we report 17 novel mutations in *RDH12*. To the best of our knowledge, this is the first study associating the clinical presentation with casual mutations in *RDH12* in a large cohort.

## Methods

### Patient selection

Patients with nonsyndromic autosomal recessive LCA or EORD were ascertained from the medical retina clinics of Moorfields Eye Hospital, London. All patients involved in this study provided written consent as part of a research project approved by the local research ethics committee. All investigations were conducted in accordance with the principles of the Declaration of Helsinki.

### Clinical evaluation

All patients underwent age-appropriate assessment of visual acuity on a LogMAR scale and funduscopy. Retinal imaging, including color fundus photography (Topcon TRC 501A retinal camera; Topcon Corporation, Tokyo, Japan), high-resolution spectral domain optical coherence tomography (SD-OCT; Spectralis spectral domain OCT scanner; Heidelberg Engineering, Heidelberg, Germany) or time-domain OCT (TD-OCT; Stratusoct Model 3000 Scanner; Zeiss Humphrey Instruments, Dublin, CA), and retinal autofluorescence (AF) imaging using a confocal scanning laser ophthalmoscope (Zeiss Prototype; Carl Zeiss, Oberkochen, Germany) was performed where nystagmus did not preclude image acquisition and in those who were old enough to cooperate. Electrophysiology had often been previously performed elsewhere, but in those patients who had not undergone previous testing, full field electroretinography and pattern electroretinography were performed. In adults and older children, these were performed using gold foil recording electrodes according to International Society for Clinical Electrophysiology of Vision (ISCEV) standards [34,35]. A modified protocol using orbital surface electrodes was used in infants and younger children, as previously described [34-38].

### DNA collection

Blood samples were collected in EDTA tubes. DNA was extracted using a Nucleon Genomic DNA extraction kit (BACC2; Tepnel Life Sciences, UK) or a Puregene kit (Invitrogen, Glasgow, UK) following the manufacturer’s instructions.

### Apex chip

Genomic DNA from 389 unrelated affected patients were sent to Asper Ophthalmics (Tartu, Estonia) for analysis using the LCA APEX chip, as described previously [39,40]. Samples in which mutations were identified in other LCA genes were excluded from further study. Much of this work has been published elsewhere [39,41-44].

### Autozygosity scan

A full genome-wide autozygosity scan was performed using all available members in families 9, 10, and 12. Samples were analyzed using the Affymetrix Gene Chip Human Mapping 50K XbaI array following the manufacturer’s instructions (Affymetrix, Santa Clara, CA). Detailed methodology for genotyping using the GeneChip array has been previously described [45]. Genotypes for single nucleotide polymorphisms (SNPs) were called by the GeneChip DNA Analysis Software (GDAS v3.0; Affymetrix). A macro was written in Visual Basic within the Microsoft Excel (Microsoft, Redmond, WA) program to detect genomic regions with a shared haplotype.

### Screening of *RDH12* by direct sequencing

Primers were used to amplify the seven coding exons and intron-exon boundaries of *RDH12* (Table 1). All PCRs were performed in a total volume of 30 μl containing 200 μM dNTPs (VH Bio, Gateshead, UK), 20 μM of each primer, 1X reaction buffer including 1.5 mM MgCl_2_ (VH Bio) with 1 unit of Moltaq (VHBio) and 100 ng of DNA. PCR was performed on a PTC200 DNA engine thermal cycler (Bio-Rad, Hemel Hempstead, UK).

**Table 1:** Primers and PCR conditions used in screening *RDH12* in this cohort.

**Exon**	**Primer**	**PCR annealing (°C)**	**Size of fragment (bp)**
Exon 1F	TTTCCCCACATTCTCTTTGCC	54	517
Exon 1R	TCCACCATGGTATCCACAACACC		
Exon 2F	TAACGTATCTTAGTGTGAGCTCG	54	306
Exon 2R	TCCTTGAATTTCTAGTCAGAGC		
Exon 3F	TCACTCTACCGTTGAAGGATGG	54	405
Exon 3R	TGTGGCAGAACCTGTCTAGTGG		
Exon 4F	ATAGTTATTGAGTGCTGAGGC	54	459
Exon 4R	TAGACTGATCAGGAGAGGTAC		
Exon 5F	TCAGACCAAACTGACCATTAGAG	54	460
Exon 5R	TGACACGTGCATGTTTGACAGCC		
Exon 6F	TGGTACCTGCTGAATCCTGGG	54	434
Exon 6R	ACCTGGATTGCATCATCAGGC		
Exon 7F	TTAGTTTCTTTGAGTCTGGC	54	884
Exon 7R	TGATTTGTTCCATTTCTCTCC		

PCR products were visualized on a 2% agarose gel containing 0.05% ethidium bromide. The products were cleaned using multiscreen PCR filter plates (cat. no. LSKMPCR10; Millipore, Watford, UK) before sequencing. PCR products were sequenced directly using the ABI Prism Big Dye terminator kit V3.1 (Life technologies, Carlsbad, CA) in a 10 μl reaction. Samples were purified using the Montage cleanup kit (cat. no. LSK509624; Millipore) before being run on an Applied Biosystems 3730 DNA Sequencer.

Analysis of electropherograms was performed by hand and using the DNA sequence analysis software Lasergene V8.1 (DNASTAR, Madison, WI). Identified mutations were confirmed bidirectionally and then checked in family members for segregation with disease. Novel missense mutations were checked in at least 100 control DNA chromosomes (European Collection of Cell Cultures and ethnically matched DNA samples). Missense mutations were analyzed using three software prediction programs: Sorting Intolerant from Tolerance (SIFT) [46], PolyPhen-2 algorithm [47], and pMUT [48].

## Results

### Mutational analysis

*RDH12* mutations (Table 2) were identified in 32 individuals from 29 families. Using the Asper Ophthalmic LCA chip on 389 patients with LCA/EORD, 11 patients were identified, with at least one mutation in *RDH12*. Direct DNA sequencing confirmed these changes and identified a second *RDH12* mutation in all of them, six of which are novel. Autozygosity mapping and subsequent direct sequencing of *RDH12* identified two more families with novel homozygous mutations (families 10 and 12). Direct sequencing was also performed on 210 LCA/EORD patients who had previously been screened across the Asper LCA chip with either no hit or with one hit in a gene. This identified four more patients with mutations in *RDH12* and five novel mutations.

**Table 2:** Results of *RDH12* mutational analysis.

**Family**	**Method of identification**	**Ethnic origin**	**Consang**	**Mutation Type**	**Mutations**	**Reference**
1	Asper	BC	No	Het	c.295C>A, p.L99I; c.883C>T, p.R295X	[32]
2	Asper	GM	Yes	Hom	c.601T>C, p.C201R	[33]
3	Asper	BC	No	Het	c.715C>T, p.R239W; c.806_810 del 5bp, p.A269AfsX1	[32]; [12]
4	Asper	BC	No	Het	c.700G>C, p.V233L; c.806_810 del 5bp,p.A269AfsX1	Novel to this study [12];
5	Asper	BC	No	Het	c.316 C>T, p.R106X; c.806_810 del 5bp,p.A269AfsX1	Novel to this study [12];
6	Asper	BC	No	Het	c.451C>G, p.H151D; c.806_810 del 5bp,p.A269AfsX1	[32]; [12]
7	Asper	BC	No	Hom	c.146C>A, p.T49K	Novel to this study
8	Asper	B	Yes	Hom	c.193C>T, p.R65X	[31]
9	Asper	OC	No	Het	c.506G>A p. R169Q; c.57_60del, p.P20del	Novel to this study
10	Asper	OC	No	Het	c.209G>A, p.C70Y ; c.806_810del5bp,p.A269AfsX1	Novel to this study [12];
11	Asper	BC	No	Het	c.144 C>T, p.R62X; c.806_810del5bp, p.A269AfsX1	[12]
12	Affymetrix	KI	Yes	Hom	c.599A>G, p.Y200C	Novel to this study
13	Affymetrix	BC	Yes	Hom	c.454T>A, p.F152I	Novel to this study
14	Affymetrix/ phenotype	I	No	Het	c.250C>T, p.R84X; c.381_delA, p.G127GfsX1	Novel to this study
15	Direct Seq	A	Yes	Hom	c.609C>A, p.S203R	Novel to this study
16	Direct Seq	P	Yes	Hom	c.506G>A, p.R169Q	Novel to this study
17	Direct Seq	BC	No	Het	c.505C>T, p.R169W; c.525C>T, p.S175L	Novel to this study [54];
18	Direct seq	PD	No	Het	c.448+1g>a; c.698insGT, p.V233VfsX45	Novel to this study
19	Phenotype	P	Yes	Hom	c.619A>G, p.N207D	Novel to this study
20	Phenotype	GM	Yes	Hom	c.601T>C, p.C201R	[33]
21	Phenotype	P	Yes	Hom	c.506G>A, p.R169Q	Novel to this study
22	Phenotype	GM	Yes	Hom	c.601T>C, p.C201R	[33]
23	Phenotype	GH	Unknown	Hom	c.146C>T, p.T49M	[12]
24	Phenotype	SA	Yes	Hom	c.609C>A, p.S203R	Novel to this study
25	Phenotype	KI	No	Hom	c.379G>T, p.G127X	[31]
26	Phenotype	GM	Yes	Hom	c.601T>C, p.C201R	[33]
27	Phenotype	OC	No	Het	c.481C>T,p.R161W ; c.714insC, p.V238VfsX34	Novel to this study
28	Phenotype	OC	Yes	Hom	c.609C>A p.S203R	Novel to this study
29	Phenotype	OC	No	Het	c.481C>T,p.R161W ; c.806_810del5bp, p.A269AfsX1	Novel to this study [12];

Table showing the results of the mutational analysis in our cohort. Mutation type; Hom – homozygous mutation, Het – heterozygous mutation. Ethnic origin key - BC – British Caucasian, OC – Other Caucasian, GM – Gujurati muslim, GH - Gujurati Hindu, A – Afghanistan, P- Pakistani, I – Indian, B –Bangladeshi, KI-Kurdistani Iraqi, SA - Saudi Arabian DP - 1/2 Portuguese 1/2 Dominican Republic

Twelve additional patients with EORD or autosomal recessive retinitis pigmentosa with a phenotype consistent with *RDH12* deficiency underwent *RDH12* screening. All had mutations in *RDH12*, with four more novel mutations being identified.

**Figure 1:**
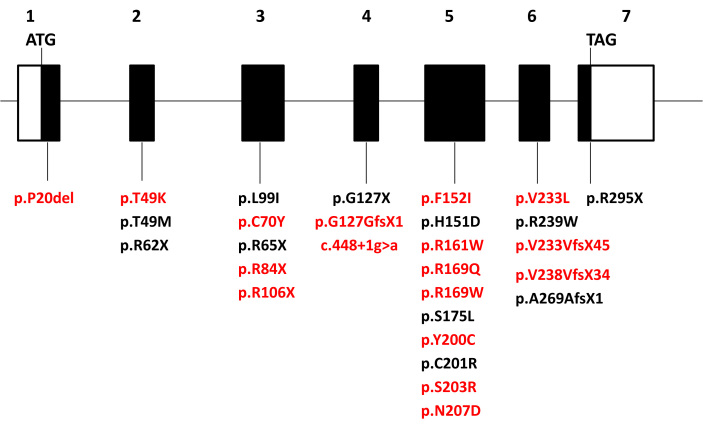
*RDH12* gene structure showing the locations of the mutations identified in this study. Novel mutations are shown in red.

Nine of 28 mutations identified in this study were located in exon 5 (Figure 1). All 17 novel mutations were absent in 100 ECACC controls or in 50 Asian controls. Where DNA samples from parents and unaffected siblings were available, further analysis demonstrated that the disease segregated with the mutations.

Analysis of all identified missense mutations using in silico methods are shown in Table 3. All three programs identified the p.C70Y, p.R169Q, p.R169W, p.Y200C, and p.R239W mutations as being intolerant or damaging to the protein. For all of the missense mutations, at least one of the programs considered the protein change to be significant.

**Table 3:** In silico analysis of identified *RDH12* missense variants.

		**SIFT**		**Polyphen-2**		**pMUT**		
**Mutation**	**Exon**	**Prediction**	**Tolerance index**	**Prediction**	**Human Var score**	**NN output**	**Reliability**	**Prediction**
**p.T49M**	**2**	Intolerant	0	PRD	0.951	0.4152	1	Neutral
p.T49K*	**2**	Intolerant	0.01	POS	0.888	0.6188	2	Pathological
p.C70Y*	**3**	Intolerant	0	PRD	0.998	0.9223	8	Pathological
**p.L99I**	**3**	Intolerant	0	PRD	0.991	0.1072	7	Neutral
**p.H151D**	**5**	Intolerant	0.01	PRD	0.992	0.3323	3	Neutral
p.F152I*	**5**	Intolerant	0	PRD	0.968	0.2127	5	Neutral
**p.R161Q**	**5**	Tolerant	0.38	Benign	0.018	0.513	0	Pathological
p.R161W*	**5**	Tolerant	0.18	POS	0.798	0.7723	5	Pathological
p.R169Q*	**5**	Intolerant	0	PRD	0.997	0.5161	0	Pathological
p.R169W*	**5**	Intolerant	0	PRD	0.999	0.8159	6	Pathological
**p.S175L**	**5**	Intolerant	0	PRD	0.997	0.2495	5	Neutral
**p.Y200C**	**5**	Intolerant	0	PRD	0.998	0.5467	0	Pathological
**p.C201R**	**5**	Tolerant	0.1	POS	0.769	0.5209	0	Pathological
p.S203R*	**5**	Intolerant	0	PRD	0.998	0.3381	3	Neutral
**p.N207D**	**5**	Intolerant	0.01	PRD	0.994	0.1661	6	Neutral
**p.V233L**	**6**	Intolerant	0.02	PRD	0.931	0.1899	6	Neutral
p.R239W	6	Intolerant	0	PRD	0.998	0.9122	8	Pathological

Changes highlighted by an asterisk are novel missense mutations identified in this study. SIFT results are reported to be tolerant if tolerance index ≥0.05 or intolerant if tolerance index <0.05. Polyphen-2 appraises mutations qualitatively as Benign, Possibly Damaging (POS) or Probably damaging (PRD) based on the model's false positive rate. pMUT is based on the use of different kinds of sequence information to label mutations, and neural networks to process this information NN=neural network values from 0 to 1. >0.5 is predicted as a disease associated mutation. Reliability=values 0–9. >5 is the best prediction.

In total, 28 different alleles in 29 families from various ethnic origins were identified (Table 2). Twelve families were consanguineous, and they harbored homozygous mutations. Two other families also had homozygous mutations, even though they did not report consanguinity. The most common mutation identified was p.C201R (8/58 alleles, 14%). Overall, missense mutations were the most prevalent mutation identified, affecting 38/58 alleles (65%). Nonsense mutations accounted for 8/58 (14%), and frameshift mutations affected 10/58 alleles (17%). The remainder of mutations consisted of a deletion of a codon (2%), and a splice site mutation (2%). Only one coding SNP was identified, rs17852293 (c.482G>A, p.R161Q), located in exon 5.

### Clinical phenotype

**Figure 2:**
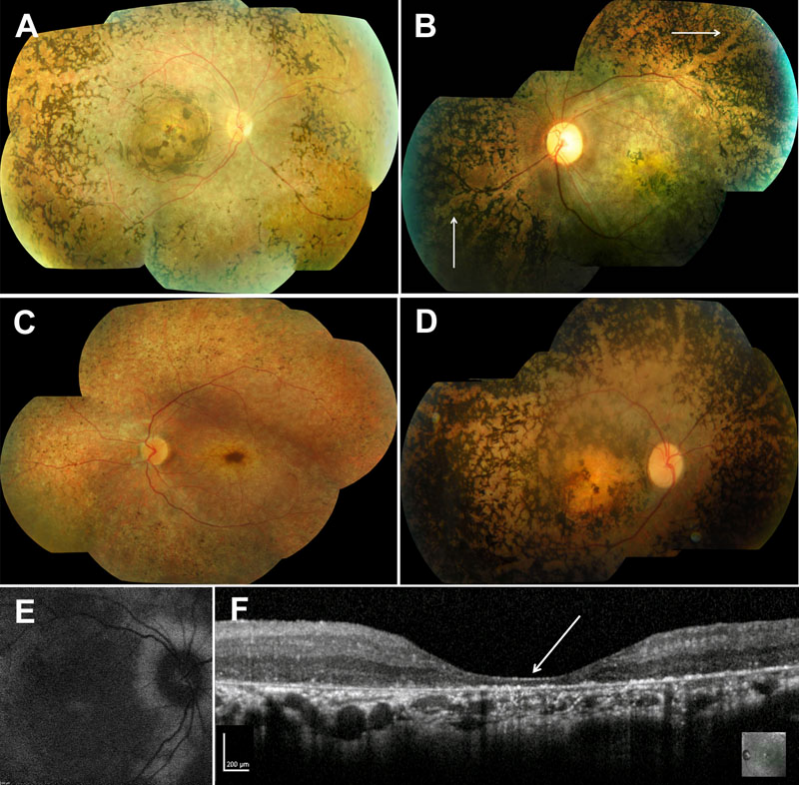
Phenotype associated with retinal dehydrogenase 12 (*RDH12*) retinopathy. **A**: Fundus appearance in adults and older children shows dense intraretinal pigment migration, severe retinal pigment epithelium atrophy, and arteriolar attenuation, with a severe atrophic pigmentary maculopathy (family 12, age 20 years). **B**: Para-arteriolar sparing of the intraretinal pigmentation was evident in six of 32 patients (white arrows, family 22, age 17.5 years). **C**: In children, retinal pigment epithelium atrophy with macular atrophy and minimal intraretinal pigmentation predominated (family 17, age 8.5 years). **D**: Macular atrophy was often associated with striking yellow deposits (family 3, age 27 years). **E**: No detectable macular autofluorescence was visible on fundus autofluorescence imaging, corresponding to the severe macular atrophy (family 11, age 11). **F**: Spectral domain optical coherence tomography demonstrated severe macular thinning, excavation, and distortion of the laminar architecture (white arrow, family 22, age 17.5 years).

Appendix 1 summarizes the clinical features of the 32 patients. Twenty-one patients (66%) presented with reduced vision. Nyctalopia (6/32) and visual field constriction (7/32) were predominant features. Twenty-nine patients reported loss of vision that was slowly progressive by age five years. Interestingly, 11 patients reported that their vision dramatically deteriorated further and were able to specify the age at which this had occurred, a median age of 26 years. Fundus examination in adults and older children revealed characteristic dense intraretinal pigment migration throughout the retina that typically approached the macula from the equator in a concentric manner, with severe RPE atrophy and arteriolar attenuation (Figure 2A). The pigmentation showed “para-arteriolar sparing” in seven patients (Figure 2B). In the younger patients (6/32, age range 5–18 years), widespread RPE atrophy was the predominant feature, with pigment migration, when present, being confined to the retinal periphery (Figure 2C). Macular atrophy was present in all cases and was associated with striking yellow deposits in 18 patients (56%; Figure 2D). AF imaging in 10 of 13 patients failed to detect any macular AF (Figure 2E), corresponding to the severe macular atrophy. The youngest patients to undergo AF imaging had overall reduced levels of macular AF but also had a hyperautofluorescent signal at the fovea (families 6, 11, and 17; age range of 5–11 years).

Ten of 13 patients underwent either Stratus OCT (6/13) or SD-OCT (7/13) imaging, which showed marked macular thinning (Figure 2F). The respective average adult foveal thicknesses observed with TD-OCT and SD-OCT were 133 µm and 56 µm (normal adult mean values: 144 µm and 228 µm [49]). In the adults who underwent SD-OCT imaging, there was marked macular excavation, severe retinal thinning, and loss of the laminar architecture (6/7 patients; Figure 2F). OCT imaging of the three youngest patients, in whom the macula was better preserved on funduscopy, demonstrated a mean foveal thickness of 167 µm (TD-OCT, families 6 and 11) and 114 µm (SD-OCT, family 17), with some preservation of the laminar architecture.

Electroretinography was performed at our institution on nine patients (age range of 2–22 years). This showed undetectable or severely attenuated rod and cone responses, demonstrating severe generalized retinal dysfunction from a very young age. This included five of the seven children below age 16 who otherwise had relatively preserved visual acuities.

## Discussion

This report on the mutational analysis and detailed description of the phenotype in a cohort of 32 patients with *RDH12* mutations represents the largest such series to be studied to date. Seventeen novel mutations are described.

The majority of the variants identified were missense mutations, with only one SNP found. Several mutations occurred more than once in the present cohort. The most common mutation, occurring in 14% of alleles, was p.C201R, which was found to be homozygous in all patients of Gujurati Indian descent. This mutation has been previously reported in one patient of Indian ancestry [33] and may represent a founder mutation in this population. The p.A269AfsX1 mutation (indentified in 12% of alleles) was found in the compound heterozygous state with another mutation in patients who were all of British Caucasian descent. This mutation was originally described in a German male in the homozygous state [12], making this a northern European mutation. Exon 5 appears to be a mutational hotspot with 9/28 mutations located in it. Therefore, screening of exon 5 in a large cohort of patients could be a first step in the identification of *RDH12* mutations. The novel variant p.R161W affects the same codon as the only SNP seen in the screening of this cohort, rs17852293 (p.R161Q*).* In silico analysis of this variant was inconclusive, but it has been considered in this paper as a potential disease variant due to its being found in the compound heterozygous state with a frameshift mutation in families 27 and 29.

The characteristic phenotype associated with *RDH12* retinopathy comprises early-onset visual loss between birth and 5 years of age (78% in the present cohort). The visual loss was progressive, leading to severe visual loss in adulthood. The subjective symptoms of nyctalopia and visual field constriction were not frequently reported at the time of presentation, but were commonly reported later in the disease course. One seldom reported feature was dramatic visual deterioration at a later age [30,31], revealed by 11 patients in this study. Cataract, a frequent feature of severe retinal degeneration [12,31,33], was present in approximately 50% of adult patients.

The defining features of *RDH12* retinopathy were present upon fundus examination. Widespread RPE atrophy predominates at a younger age, with minimal intraretinal pigment migration. Despite the presence of maculopathy at this stage, the central foveal thickness and foveal architecture may still be preserved. As patients age, there is striking intraretinal pigmentation, possibly with para-arteriolar sparing, accompanied by severe pigmentary maculopathy, characteristic yellow macular deposits, and macular excavation. There is little or no autofluorescence at the macula, in keeping with severe macular atrophy. SD-OCT imaging demonstrated severe macular thinning and the excavation and loss of the foveal laminar architecture. These SD-OCT data are in agreement with previous studies using TD-OCT imaging [27].

No genotype-phenotype correlation could be identified for age of onset, age at diagnosis, presenting features, refractive error, or visual acuity.

There are limited data regarding the histopathology of intraretinal pigment migration (or bone spicule pigmentation). A recent study of the rhodopsin knockout (*rho^−/−^*) mouse, a murine model of human retinitis pigmentosa, demonstrated that the migration of RPE cells along blood vessels within the inner retina is triggered by the close approximation and direct contact of the inner retinal vessels with the RPE [50]. This is a consequence of the loss of photoreceptor cells and subsequent reduction of retinal thickness, which causes an approximation of the inner retinal layers with the RPE. Subsequent bone spicule pigmentation occurs as pigmented cell clusters form over most of the retinal capillaries except for the large surface vessels. This may explain the distribution of the intraretinal pigment in *RDH12* retinopathy, and possibly the observation of para-arteriolar sparing, which is also a feature in *CRB1* disease [11]. The severe macular atrophy in the *RDH12* phenotype is also consistent with the increased susceptibility at the macula to light-induced photoreceptor apoptosis that has been observed in *RDH12* knockout mice [24], supporting evidence for the unique role of this protein in the photoreceptor inner segment as a retinoid regulator. The disease mechanism is also not solely dependent upon loss of enzymatic function. It has been shown that some missense mutations in *RDH12* retain enzymatic function but are believed to undergo accelerated degradation [51].

*RDH12* mutations account for 7% of disease in the cohort of patients with LCA or EORD at this institution, similar to the frequency of *CRB1* mutations in the same group of patients [52]. This is higher than the previously published 2.7% [53]. This higher incidence and the number of novel changes may reflect the use of the APEX microarray to identify known changes and the use of detailed phenotypic data. Currently there is no treatment for *RDH12* associated disease. However, would a future treatment become available, the optimum time of intervention should be at a young age, before the onset of severe retinal pigmentation.

## Appendix 1. Clinical features of patients identified with *RDH12* mutations.

To access the data, click or select the words “Appendix 1.” This will initiate the download of a compressed (pdf) archive that contains the file. Abbreviations: CF represents counting fingers; DS represents diopter sphere; EE represents either eye; F represents female; HM represents hand movements; LE represents left eye; M represents Male, N/A represents not available; PCIOL represents posterior chamber intra ocular lens; PL represents perception of light; PSCLO represents posterior subcapsular lens opacification; RE represents right eye; VF represents visual field.
